# Evaluation of Plasma Biomarkers for A/T/N Classification of Alzheimer Disease Among Adults of Caribbean Hispanic Ethnicity

**DOI:** 10.1001/jamanetworkopen.2023.8214

**Published:** 2023-04-20

**Authors:** Lawrence S. Honig, Min Suk Kang, Annie J. Lee, Dolly Reyes-Dumeyer, Angel Piriz, Belisa Soriano, Yahaira Franco, Zoraida Dominguez Coronado, Patricia Recio, Diones Rivera Mejía, Martin Medrano, Rafael A. Lantigua, Andrew F. Teich, Jeffrey L. Dage, Richard Mayeux

**Affiliations:** 1 Taub Institute for Research on Alzheimer’s Disease and the Aging Brain, Vagelos College of Physicians and Surgeons, Columbia University, New York, New York; 2 G. H. Sergievsky Center, Vagelos College of Physicians and Surgeons, Columbia University, New York, New York; 3 Universidad Pedro Henríquez Urena, Santo Domingo, Dominican Republic; 4 Clínica Corominas, Santiago, Dominican Republic; 5 Clínica Gregorio Hernandez, Puerto Plata, Dominican Republic; 6 Center for Diagnosis, Advanced Medicine and Telemedicine, Santo Domingo, Dominican Republic; 7 Pontíficia Universidad Católica Madre y Maestra, Santiago, Dominican Republic; 8 Department of Medicine, Vagelos College of Physicians and Surgeons, New York Presbyterian Hospital, Columbia University, New York, New York; 9 Department of Neurology, Vagelos College of Physicians and Surgeons, New York Presbyterian Hospital, Columbia University, New York, New York; 10 Department of Pathology and Cell Biology, Vagelos College of Physicians and Surgeons, Columbia University, New York, New York; 11 Department of Neurology, Indiana University School of Medicine, Indianapolis

## Abstract

**Importance:**

Cerebrospinal fluid (CSF) and plasma biomarkers can detect biological evidence of Alzheimer disease (AD), but their use in low-resource environments and among minority ethnic groups is limited.

**Objective:**

To assess validated plasma biomarkers for AD among adults of Caribbean Hispanic ethnicity.

**Design, Setting, and Participants:**

In this decision analytical modeling study, adults were recruited between January 1, 2018, and April 30, 2022, and underwent detailed clinical assessments and venipuncture. A subsample of participants also consented to lumbar puncture. Established CSF cut points were used to define AD biomarker-positive status, allowing determination of optimal cut points for plasma biomarkers in the same individuals. The performance of a panel of 6 plasma biomarkers was then assessed with respect to the entire group. Data analysis was performed in January 2023.

**Main Outcomes and Measures:**

Main outcomes were the association of plasma biomarkers amyloid-β 1-42 (Aβ42), amyloid-β 1-40 (Aβ40), total tau (T-tau), phosphorylated tau181 (P-tau181), glial fibrillary acidic protein (GFAP), and neurofilament light chain (NfL) with AD diagnosis. These biomarkers allow assessment of amyloid (A), neurofibrillary degeneration (T), and neurodegeneration (N) aspects of AD. Statistical analyses performed included receiver operating characteristics, Pearson and Spearman correlations, *t* tests, and Wilcoxon rank-sum, chi-square, and Fisher exact tests.

**Exposures:**

Exposures included age, sex, education, country of residence, apolipoprotein-ε4 (*APOE-ε4*) allele number, serum creatinine, blood urea nitrogen, and body mass index.

**Results:**

This study included 746 adults. Participants had a mean (SD) age of 71.0 (7.8) years, 480 (64.3%) were women, and 154 (20.6%) met clinical criteria for AD. Associations were observed between CSF and plasma P-tau181 (*r* = .47 [95% CI, 0.32-0.60]), NfL (*r* = 0.57 [95% CI, 0.44-0.68]), and P-tau181/Aβ42 (*r* = 0.44 [95% CI, 0.29-0.58]). For AD defined by CSF biomarkers, plasma P-tau181 and P-tau181/Aβ42 provided biological evidence of AD. Among individuals judged to be clinically healthy without dementia, biomarker-positive status was determined by plasma P-tau181 for 133 (22.7%) and by plasma P-tau181/Aβ42 for 104 (17.7%). Among individuals with clinically diagnosed AD, 69 (45.4%) had plasma P-tau181 levels and 89 (58.9%) had P-tau181/Aβ42 levels that were inconsistent with AD. Individuals with biomarker-negative clinical AD status tended to have lower levels of education, were less likely to carry *APOE-ε4* alleles, and had lower levels of GFAP and NfL than individuals with biomarker-positive clinical AD.

**Conclusions and Relevance:**

In this cross-sectional study, plasma P-tau181 and P-tau181/Aβ42 measurements correctly classified Caribbean Hispanic individuals with and without AD. However, plasma biomarkers identified individuals without dementia with biological evidence of AD, and a portion of those with dementia whose AD biomarker profile was negative. These results suggest that plasma biomarkers can augment detection of preclinical AD among asymptomatic individuals and improve the specificity of AD diagnosis.

## Key Points

**Question:**

Can plasma biomarker analytes be used in a low-resource community to improve clinical accuracy in diagnosing Alzheimer disease (AD)?

**Findings:**

In this decision analytical modeling study of 746 Caribbean Hispanic individuals from the Dominican Republic and New York, a panel of plasma biomarkers, including phosphorylated tau181 (P-tau181) and the ratio of P-tau181 to amyloid-β Aβ42, identified biological evidence of AD. A proportion of asymptomatic individuals without dementia had biomarker evidence of AD and may be presymptomatic, while a proportion of affected individuals with dementia lacked biomarker evidence of AD and may have other dementia disorders.

**Meaning:**

These findings suggest that plasma biomarkers can improve the specificity of the clinical diagnosis of AD and can detect biological evidence of the disease in asymptomatic individuals in a low-resource environment where other types of diagnostic procedures are limited.

## Teaser

This decision analytical modeling study evaluates the value of validated plasma biomarkers for Alzheimer disease for application of the A/T/N system among adults of Caribbean Hispanic ethnicity.

## Introduction

Cerebrospinal fluid (CSF) biomarkers have shown excellent sensitivity and specificity when cerebral β-amyloidosis on positron emission tomography (PET) or autopsy-confirmed Alzheimer disease (AD) are used as standard references.^1,2,3,4,5,6,7^ The goal of these biomarkers is to meet the required elements of the A/T/N system, which divides AD biomarkers into 3 pathophysiologic categories as follows: A refers to a β-amyloid biomarker (amyloid PET or CSF amyloid-β 1-42 [Aβ42] or CSF amyloid-β 1-42 to amyloid-β 1-40 [Aβ40] ratio), T to a tau biomarker (CSF phosphorylated tau [P-tau] or tau PET), and N to a neurodegeneration or neuronal injury biomarker ([^18^F]-fluorodeoxyglucose–PET, structural magnetic resonance imaging [MRI], or CSF total tau [T-tau] or CSF neurofilament light chain [NfL]).^8^ More recently, plasma biomarkers have emerged that appear to rival CSF-based biomarkers at detecting underlying pathologic evidence of AD consistent with the A/T/N system.^9,10,11,12,13,14,15,16^ However, these studies—both clinic and community based—have generally consisted of non-Hispanic White individuals with high levels of education and low frequencies of comorbidities.^9,10,11,12,17^

The use of plasma-based biomarkers in low-resource communities would greatly facilitate the diagnosis of AD worldwide and allow for application of the A/T/N classification. Such communities often lack highly trained providers to conduct lumbar puncture, diagnostic tools such as structural or functional brain imaging, and the ability to confirm AD by autopsy at the time of death. In addition, some social determinants, such as low educational attainment, may complicate the diagnosis of dementia.^18^

In this study, we evaluated the performance of plasma-based biomarkers in a low-resource environment in which no PET imaging capability exists and there is limited autopsy availability. The goal of this investigation was to determine how to assess the value of a panel of plasma biomarkers for application of the A/T/N system among healthy individuals and those with a clinical diagnosis of AD in a research or clinical setting. We were particularly interested in the added objective specificity that biomarkers may contribute to the clinical diagnosis for individuals of Caribbean Hispanic ethnicity.

## Methods

### Participants

For this decision analytical modeling study of aging and dementia^19^ in Caribbean Hispanic individuals, participants provided written informed consent under protocols approved by the Columbia University Irving Medical Center Institutional Review Board and the National Health Bioethics Committee of the Dominican Republic. This study followed the Consolidated Health Economic Evaluation Reporting Standards (CHEERS) reporting guideline.

Participants were recruited between January 1, 2018, and April 30, 2022, using local newspaper and radio advertisements and referrals from clinics from villages and cities throughout the Dominican Republic and from the Washington Heights area of northern Manhattan. They underwent a medical interview, medical and neurological examination, neuropsychological testing battery, and venipuncture for plasma and DNA. We were able to perform both CSF and plasma measurements in a subsample of patients. We used laboratory-specific, single-molecule array (Simoa) CSF cut points for AD to determine plasma cut points in the same individuals. We then examined the performance of measured plasma biomarkers Aβ42, Aβ40, T-tau, P-tau181, glial fibrillary acidic protein (GFAP), and NfL in individuals with a clinical diagnoses of AD or normal aging. Clinical diagnoses were established in a consensus conference at which a panel including a neuropsychologist, a neurologist, and an internist with expertise in dementia and geriatrics (without access to biomarker data) reviewed all clinical data (Table 1).

**Table 1.** Demographic Characteristics and Clinical Diagnoses of Study Participants^a^

Characteristic	Participant group (N = 746)		*P* value
	With clinical AD (n = 154)	Without dementia (n = 592)	
Age, mean (SD), y	76.4 (8.2)	69.7 (7.2)	9.6 × 10^−21^
Sex			
Women	99 (64.3)	381 (64.4)	.99
Men	55 (35.7)	211 (35.6)	.99
Education level, mean (SD), y	3.4 (4.1)	5.6 (4.7)	5.6 × 10^−9^
Dominican Republic residence	136 (88.3)	542 (91.6)	.21
Clinical Dementia Rating scale score			
0	0	415 (70.1)	NA
0.5	0	177 (29.9)	NA
≥1	154 (100)	0	NA
*APOE-ε4* (>1 allele)^b^	51/118 (43.2)	166/431 (38.5)	.34
Laboratory value, mean (SD)			
Serum creatinine, mg/dL	0.9 (0.3)	0.9 (0.3)	.06
Serum BUN, mg/dL	14.8 (5.4)	14.3 (5.1)	.43
BUN/creatinine ratio	16.3 (5.2)	16.4 (5.3)	.85
Body mass index, mean (SD)^c^	26.7 (5.7)	27.7 (5.1)	.009

Abbreviations: AD, Alzheimer disease; *APOE-ε4*, apolipoprotein-ε4; BUN, blood urea nitrogen; NA, not applicable.

SI conversion factors: To convert creatinine to μmol/L, multiply by 88.4. To convert BUN to mmol/L, multiply by 0.357.

^a^ Unless indicated otherwise, values are reported as No. of participants (%).

^b^ Values were missing for 38 patients with clinical AD and 161 without dementia.

^c^ Calculated as weight in kilograms divided by height in meters squared.

### Sample Collection

Blood for plasma was collected in dipotassium ethylenediaminetetraacetic acid tubes and centrifuged at 2000*g* for 15 minutes at 4 °C within 2 hours after collection. Plasma was aliquoted in polypropylene tubes, frozen, and stored at −80 °C. Blood for DNA extraction was also collected. Apolipoprotein E (*APOE*) genotyping was performed at LGC Genomics and CD Genomics. Cerebrospinal fluid was obtained with a standard aseptic technique, distributed into aliquots of 400 μL each in polypropylene tubes, frozen, and stored at −80 °C.

### Biomarker Assays

We performed CSF biomarker assays using the Simoa SR-X platform^1,20^ and plasma biomarker assays using the Simoa HD-X platform (both Quanterix). Samples were assayed in duplicate per the package insert instructions using the following Quanterix kits: Neurology 3-Plex A (catalog No. 101995) for Aβ42, Aβ40, and T-tau; pTau-181 V2 Advantage (catalog No. 103714) for P-tau181; and Neurology 2-Plex B (catalog No. 103520) for GFAP and NfL. Ratios of Aβ42/Aβ40, T-tau/Aβ42, and P-tau181/Aβ42 were calculated. Cerebrospinal fluid positivity for AD was determined using the CSF P-tau181/Aβ42 optimal cut point of 0.223 established in our laboratory. This CSF cut point is derived from receiver operating characteristic (ROC) analysis of a validation group of combined autopsy cases (n = 20) and amyloid PET cases (n = 59) with CSF biomarkers, including Aβ40, Aβ42, T-tau, P-tau181, and NfL (using Quanterix kit 103400), measured on the SR-X system. The area under the curve (AUC) was best for P-tau181/Aβ42, at 0.88 (0.79-0.97) with a Youden index of 0.82. At the P-tau181/Aβ42 cut point of 0.22, sensitivity was 0.95 and specificity was 0.87.

### Statistical Analysis

We performed ROC analysis to determine the AUC, sensitivity, specificity, and Youden index values. Associations between plasma and CSF biomarkers were assessed with Pearson or Spearman correlation coefficients. Mean differences in continuous traits between 2 groups were assessed using independent *t* tests or nonparametric equivalent Wilcoxon rank-sum tests. Proportions of variables by 2 groups were assessed with the χ^2^ or Fisher exact test, using 2-sided tests and a threshold of *P* = .05 for significance. Normality of the data distribution was determined using the Shapiro-Wilk test. Analyses were performed using R, version 4.2.1 (R Foundation for Statistical Computing), and SPSS, version 27.0 (IBM SPSS). Data analysis was performed in in January 2023.

## Results

This decision analytical modeling study included 746 adults of Caribbean Hispanic ethnicity. Of the participants, 678 (90.9%) were from the Dominican Republic and 68 (9.1%) were from the Washington Heights area of northern Manhattan. Their mean (SD) age was 71.0 (7.8) years, and there were 480 women (64.3%) and 266 men (35.7%). A total of 154 participants (20.6%) met clinical criteria for AD.^21^ No other forms of clinical dementia were encountered, and the remaining 592 patients (79.4%) did not have clinical evidence of dementia. A subset of 127 participants (17.0%) provided CSF, and lumbar puncture was performed in these individuals; 35 (27.6%) met clinical criteria for AD.^21^

### Establishing Plasma Measurement Cut Points

Among the 127 individuals with both plasma and CSF biomarker data, there were associations between plasma and CSF levels for P-tau181 (*r* = 0.47 [95% CI, 0.32-0.60]), NfL (*r* = 0.57 [95% CI, 0.44-0.68]), and P-tau181/Aβ42 (*r* = 0.44 [95% CI, 0.29-0.58]) (*P* < .001). The correlation coefficient for plasma and CSF levels of Aβ42/Aβ40 was smaller (*r* = 0.31 [95% CI, 0.14-0.46]); plasma and CSF levels of Aβ40, Aβ42, T-tau, and T-tau/Aβ42 were not associated. Using our established laboratory CSF cut points mentioned earlier to define biological AD, we then performed an analysis of plasma biomarkers in the same individuals to determine the classification performance and optimal cut points for each plasma biomarker. The ROC curves plotted for the 5 individual plasma analytes and the 3 specified ratios are shown in the Figure. Plasma P-tau181 and P-tau181/Aβ42 emerged as the best indicators of CSF-positive AD. In P-tau181 analyses, we observed an AUC of 0.86 (95% CI, 0.78-0.94) with sensitivity of 0.89 (95% CI, 0.65-0.99), specificity of 0.78 (95% CI, 0.68-0.85), and a Youden index of 0.66, with the optimal cut point of 2.63 pg/mL. Similarly, for P-tau181/Aβ42, we observed an AUC of 0.86 (95% CI, 0.77-0.95) with sensitivity of 0.94 (95% CI, 0.73-1.00), specificity of 0.75 (95% CI, 0.65-0.83), and a Youden index of 0.69, with the optimal cut point of 0.26 (Table 2).

**Figure.**
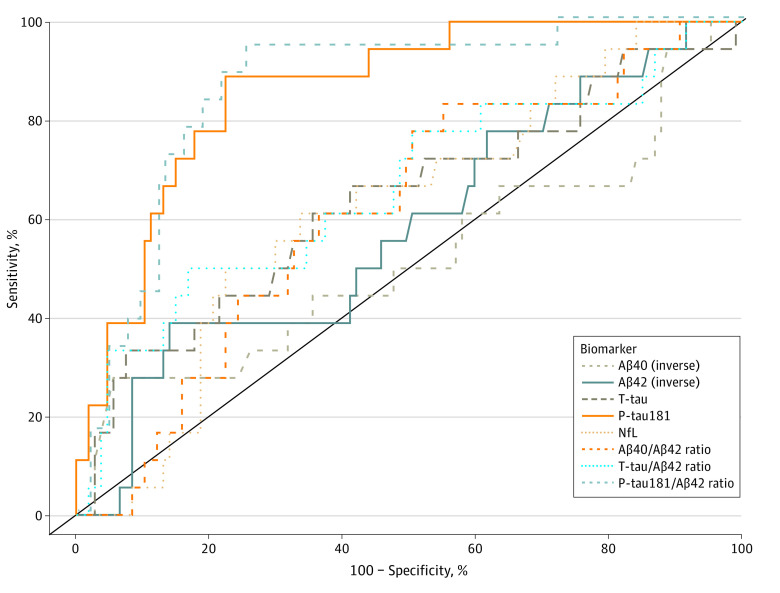
Receiver Operator Curve Analyses of Plasma Biomarker Performance in Classifying Cerebrospinal Fluid (CSF)–Supported Diagnosis of Alzheimer Disease Performance of plasma biomarkers amyloid-β 1-40 (Aβ40) (inverse), amyloid-β 1-42 (Aβ42) (inverse), total tau (T-tau), neurofilament light chain (NfL), phosphorylated tau181 (P-tau181), and ratios of Aβ40/Aβ42, T-tau/Aβ42, and P-tau181/Aβ42 in classifying CSF biomarker (P-tau181/Aβ42)–supported diagnosis of biological Alzheimer disease.

**Table 2.** Receiver Operating Curve Analyses for Plasma Analytes Based on Cerebrospinal Fluid (CSF)–Diagnosed Alzheimer Disease^a^

Plasma analyte	Area under the curve (95% CI)	Sensitivity (95% CI)	Specificity (95% CI)	Youden index	Cut point
Aβ40 (inverse), mL/pg^a^	0.52 (0.35-0.69)	0.28 (0.10-0.53)	0.94 (0.88-0.98)	0.22	0.007
Aβ42 (inverse), mL/pg	0.58 (0.44-0.73)	0.39 (0.17-0.64)	0.86 (0.78-0.92)	0.25	0.13
T-tau, pg/mL	0.64 (0.48-0.79)	0.33 (0.13-0.59)	0.93 (0.86-0.97)	0.26	2.74
P-tau181, pg/mL	0.86 (0.78-0.94)	0.89 (0.65-0.99)	0.78 (0.68-0.85)	0.67	2.63
NfL, pg/mL	0.62 (0.49-0.75)	0.50 (0.26-0.74)	0.78 (0.68-0.85)	0.28	21.10
GFAP, pg/mL	0.82 (0.71-0.92)	0.94 (0.73-1.00)	0.58 (0.48-0.67)	0.52	133
Aβ40/Aβ42	0.62 (0.48-0.75)	0.83 (0.59-0.96)	0.45 (0.35-0.55)	0.28	19.40
T-tau/Aβ42	0.66 (0.51-0.81)	0.50 (0.26-0.74)	0.83 (0.75-0.90)	0.33	0.23
P-tau181/Aβ42	0.86 (0.77-0.95)	0.94 (0.73-1.00)	0.75 (0.65-0.83)	0.69	0.26

Abbreviations: Aβ40, amyloid-β 1-40; Aβ42, amyloid-β 1-42; GFAP, glial fibrillary acidic protein; NfL, neurofilament light chain; P-tau181, phosphorylated tau181; T-tau, total tau.

^a^ Reciprocal of the analyte (inverse).

### Comparison of Clinical and Biological AD by Plasma Biomarkers

We applied the plasma analyte cut points established earlier to the entire group of 746 individuals, including the 127 individuals with CSF who were similar in mean age, sex distribution, education, and recruitment origins to the other 619 individuals. Individuals with clinically diagnosed AD were notably older and had lower levels of education, but there was no difference in *APOE-ε4* frequency. Compared with individuals without dementia, those with clinical AD had significantly higher P-tau181, NfL, Aβ40, and GFAP levels (Table 3 and Table 4). No differences emerged after adjusting for blood urea nitrogen or creatinine levels, which may be due, in part, to the overall lack of severe kidney disease in the recruited individuals.

**Table 3.** Comparison of Individuals Without Dementia and Clinically Diagnosed Alzheimer Disease (AD) Defined by the Optimal Cut Point Established for P-tau181^a^

Characteristic	Participant group					
	With clinical AD			Without dementia		
	Negative (n = 69)	Positive (n = 83)	*P* value	Negative (n = 454)	Positive (n = 133)	*P* value
Age, y, mean (SD)	76.1 (8.0)	76.6 (8.4)	.22	69.2 (7.1)	71.6 (7.2)	.001
Sex						
Women	44 (63.8)	55 (66.3)	.75	289 (63.7)	89 (66.9)	.49
Men	25 (36.2)	28 (33.7)		165 (36.3)	44 (33.1)	
Education level, y	2.6 (3.2)	4.1 (4.6)	.04	5.6 (4.7)	5.3 (4.4)	.73
*APOE-ε4* allele (≥1)^b^	18/49 (36.7)	33/68 (48.5)	.20	116/329 (35.3)	47/97 (48.5)	.01
Cognitive/functional assessment						
I-ADL score^c^	5.3 (1.9)	6.1 (1.3)	.08	0.5 (1.0)	0.9 (1.6)	.025
Animal fluency	9.1 (4.3)	7.3 (4.6)	.03	13.1 (4.9)	12.4 (4.8)	.12
CFL fluency	5.3 (9.8)	6.9 (9.2)	.12	17.6 (13.9)	17.1 (12.5)	.99
SRT score^d^						
Total recall	20.0 (8.4)	16.6 (9.4)	.02	37.6 (9.9)	36.3 (8.4)	.29
Delayed recall	1.7 (1.6)	0.9 (1.6)	<.001	4.6 (1.9)	4.2 (1.9)	.047
Orientation^e^	7.5 (2.4)	6.3 (3.2)	.018	9.1 (1.3)	9.1 (1.0)	.74
Biomarker value						
Aβ40, pg/mL	261.7 (98.4)	275.4 (92.9)	.20	238.0 (70.7)	272.9 (118.9)	.003
Aβ42, pg/mL	12.9 (4.8)	12.9 (4.8)	.85	12.1 (3.7)	13.7 (6.5)	.008
Aβ40/Aβ42 ratio	21.6 (8.6)	22.1 (5.7)	.07	21.2 (18.4)	21.4 (10.7)	.51
T-tau, pg/mL	2.6 (1.2)	3.1 (1.7)	.30	2.5 (1.2)	3.1 (2.3)	.019
NfL, pg/mL	21.6 (15.3)	31.5 (25.1)	.001	15.7 (17.6)	23.9 (25.3)	1.9 × 10^−8^
GFAP, pg/mL	174.6 (154.1)	268.4 (157.4)	2.1 × 10^−6^	128.5 (80.6)	185.4 (123.1)	8.4 × 10^−10^
Laboratory value						
Serum creatinine, mg/dL	0.9 (0.2)	1.0 (0.4)	.60	0.9 (0.2)	1.1 (0.6)	8.5 × 10^−6^
Serum BUN, mg/dL	14.4 (4.3)	15.2 (6.2)	.70	13.7 (4.3)	16.4 (6.6)	<.001
BUN/creatinine ratio	16.3 (4.6)	16.1 (5.8)	.77	16.4 (5.3)	16.3 (5.3)	.99
Body mass index^f^	28.0 (6.5)	25.7 (4.6)	.049	27.8 (5.2)	27.6 (5.1)	.58

Abbreviations: Aβ40, amyloid-β 1-40; Aβ42, amyloid-β 1-42; *APOE-ε4*, apolipoprotein-ε4; BUN, blood urea nitrogen; CFL, verbal fluency sum (for letters C, F, and L); GFAP, glial fibrillary acidic protein; NfL, neurofilament light chain; P-tau181, phosphorylated tau181; T-tau, total tau.

SI conversion factors: To convert creatinine to μmol/L, multiply by 88.4. To convert BUN to mmol/L, multiply by 0.357.

^a^ Unless indicated otherwise, values are reported as No. of individuals (%). The total sample size was less than 746 due to failed measurements (7 for Aβ42 and 18 for P-tau181). Participants were compared based on clinical AD and biomarker status based on the optimal cut point for plasma P-tau181 (negative indicated below the cut point and positive indicated above the cut point). Phosphorylated-tau181 levels were not included here because they were used to define those above or below the optimal cut point.

^b^ Values were missing for 20 individuals with biomarker-negative clinical AD, 15 with biomarker-positive clinical AD, 125 without dementia and biomarker-negative status, and 36 without dementia and biomarker-positive status.

^c^ Instrumental Activities of Daily Living (I-ADL) scores range from 0 (best) to 8 (worst).

^d^ Selective Reminding Test (SRT) total and delayed recall maximum (best) scores are 72 and 12.

^e^ Orientation maximum (best) score is 10 (5 for time and 5 for place).

^f^ Calculated as weight in kilograms divided by height in meters squared.

**Table 4.** Comparison of Individuals Without Dementia and Those With Clinically Diagnosed Alzheimer Disease (AD) Defined by the Optimal Cut Point Established for P-tau181/Aβ42^a^

Characteristic	Participant group					
	With clinical AD			Without dementia		
	Negative (n = 89)	Positive (n = 62)	*P* value	Negative (n = 473)	Positive (n = 104)	*P* value
Age, y, mean (SD)	77.4 (7.9)	74.8 (8.5)	.14	69.5 (7.3)	70.4 (6.5)	.15
Sex						
Women	59 (66.3)	40 (64.5)	.82	307 (64.9)	69 (66.3)	.78
Men	30 (33.7)	22 (35.5)		166 (35.1)	35 (35.7)	
Education level, y	2.5 (3.1)	4.7 (5.0)	.004	5.6 (4.7)	5.2 (4.2)	.62
*APOE-ε4* allele (≥1)^b^	20/63 (31.7)	31/53 (58.5)	.004	117/340 (34.4)	41/77 (53.2)	.001
Cognitive/functional assessment						
I-ADL^c^	5.3 (1.7)	6.2 (1.5)	.002	0.6 (1.2)	0.7 (1.2)	.11
Animal fluency	9.1 (4.3)	6.7 (4.6)	<.001	13.0 (5.0)	12.4 (4.9)	.14
CFL fluency	5.0 (9.2)	8.0 (9.6)	.019	17.7 (13.7)	16.4 (13.3)	.36
SRT score^d^						
Total recall	20.3 (8.7)	15.2 (8.9)	9.4 × 10^−5^	37.6 (9.5)	36.0 (7.5)	.16
Delayed recall	1.7 (1.7)	0.6 (1.4)	3.5 × 10^−5^	4.6 (1.9)	4.2 (2.1)	.27
Orientation^e^	7.5 (2.5)	5.9 (3.2)	<.001	9.1 (1.3)	8.9 (1.1)	.04
Biomarker						
Aβ40, pg/mL	294.5 (101.3)	233.0 (72.6)	.001	254.6 (84.8)	209.5 (75.3)	3.0 × 10^−7^
Aβ42, pg/mL	14.9 (4.7)	10.0 (3.2)	1.0 × 10^−10^	13.3 (4.3)	8.8 (3.6)	2.8 × 10^−23^
Aβ40/Aβ42 ratio	19.9 (3.6)	24.7 (9.6)	<.001	19.6 (4.4)	29.0 (37.9)	5.4 × 10^−8^
T-tau, pg/mL	3.0 (1.5)	2.7 (1.6)	.11	2.7 (1.5)	2.5 (1.8)	.08
NfL, pg/mL	25.9 (20.5)	28.6 (23.5)	.30	17.3 (20.7)	18.9 (17.0)	.06
GFAP, pg/mL	196.0 (145.6)	269.0 (177.4)	<.001	135.1 (83.9)	172.7 (131.4)	.005
Laboratory value						
Serum creatinine, mg/dL	1.0 (0.4)	0.9 (0.2)	.06	0.9 (0.4)	0.9 (0.3)	.79
BUN, mg/dL	15.3 (5.4)	14.2 (5.5)	.18	14.2 (4.9)	14.9 (5.5)	.32
BUN/creatinine ratio	15.9 (4.4)	16.8 (6.4)	.62	16.3 (5.2)	16.9 (5.8)	.48
Body mass index^f^	27.1 (6.1)	26.2 (5.0)	.75	27.9 (5.1)	27.0 (5.2)	.06

Abbreviations: Aβ40, amyloid-β 1-40; Aβ42, amyloid-β 1-42; *APOE-ε4*, apolipoprotein-ε4; BUN, blood urea nitrogen; CFL, verbal fluency sum (for letters C, F, and L); GFAP, glial fibrillary acidic protein; NfL, neurofilament light chain; P-tau181, phosphorylated tau-181; T-tau, total tau-181.

SI conversion factors: To convert creatinine to μmol/L, multiply by 88.4. To convert BUN to mmol/L, multiply by 0.357.

^a^ Unless indicated otherwise, values are reported as No. of individuals (%). The total sample size was less than 746 due to failed measurements (7 for Aβ42 and 18 for P-tau181). Participants are compared based on clinical AD and biomarker status based on the optimal cut point for plasma P-tau181/Aβ42 (negative indicated below the cut point and positive indicated above the cut point). Phosphorylated-tau181 and P-tau181/Aβ42 levels were not included here because they were used to define those above or below the optimal cut point.

^b^ Values were missing for 26 individuals with biomarker-negative clinical AD, 9 with biomarker-positive clinical AD, 133 without dementia and biomarker-negative status, and 27 without dementia and biomarker-positive status.

^c^ Instrumental Activities of Daily Living (I-ADL) scores range from 0 (best) to 8 (worst).

^d^ Selective Reminding Test (SRT) total and delayed recall maximum (best) scores are 72 and 12.

^e^ Orientation maximum (best) score is 10 (5 for time and 5 for place).

^f^ Calculated as weight in kilograms divided by height in meters squared.

Subsequently, we compared the diagnosis of clinical AD vs biological AD based on the plasma P-tau181 and P-tau181/Aβ42 biomarkers. A total of 154 individuals (20.6%) had a clinical diagnosis of AD. Of these individuals, biological AD was identified in only 83 (54.6%) based on plasma P-tau181 and in 62 (41.1%) based on plasma P-tau181/Aβ42. Of the 592 individuals judged clinically to not have dementia, 454 (77.3%) had P-tau181 levels below the cut point and 473 (82.0%) had P-tau181/Aβ42 levels below the cut point, consistent with their clinical diagnosis. However, 133 individuals (22.7%) had P-tau181 levels above the cut point, while 104 (17.7%) had P-tau181/Aβ42 above the cut point, consistent with biological AD despite being asymptomatic. The proportions of individuals whose plasma biomarkers were consistent with their clinical diagnoses did not change substantially, even if a gray zone was established (rather than using a single cut point) excluding potentially indeterminate individuals whose biomarker levels were within 5% or 10% closest to the cut points (data not shown).

We compared individuals without dementia who had plasma biomarker-negative status (by either P-tau181 or P-tau181/Aβ42) with those without dementia who had biomarker-positive status. Individuals with biomarker-negative status were substantially older and had lower levels of other biomarkers, including Aβ40, GFAP, and NfL (Tables 3 and 4). In addition, there was little difference in cognitive measurements between the 2 groups. Compared with individuals with clinical dementia who had AD biomarker-positive status (either by P-tau181 or P-tau181/Aβ42), those with dementia and biomarker-negative status were of similar age and sex, had lower education levels, and had lower levels of neurodegenerative markers GFAP and NfL (P-tau181 only) (Tables 3 and 4). For the subgroup with CSF biomarkers, similar results were observed with lower CSF T-tau (data not shown), but the numbers were very small.

## Discussion

The results of this decision analytical modeling study suggest that using plasma biomarkers can add precision to the clinical diagnosis of AD in a low-resource community population. Like others, we observed that plasma biomarker levels, especially P-tau181 and P-tau181/Aβ42, were associated with CSF levels in the group in which both were measured. Among the larger group of Caribbean Hispanic research participants, plasma biomarkers P-tau181 and P-tau181/Aβ42, with cut points established based on analysis of the CSF subgroup, performed reasonably well in identifying biological AD. In effect, these plasma biomarkers allowed us to incorporate the A/T/N classification.^8^

Using the plasma cut points established with the 127 individuals with both CSF and plasma enabled us to compare the clinical diagnoses derived from a comprehensive cognitive and functional assessment with the biomarkers in plasma. Using the plasma biomarkers as evidence of AD pathology and defining the outcome as biological AD, we determined that 54.6% of individuals clinically diagnosed with AD had biological AD based on plasma P-tau181 and 41.1% had biological AD based on plasma P-tau181/Aβ42. Conversely, among individuals without dementia, 22.7% had P-tau181 levels above the cut point and 17.7% had P-tau181/Aβ42 above the cut point, consistent with the diagnosis of biological AD despite being asymptomatic. Interestingly, individuals with elevated P-tau181 or P-tau181/Aβ42, regardless of clinical diagnosis, were more often carriers of the *APOE-ε4* allele and had elevated GFAP levels.

Individuals with clinical AD included a substantial proportion with biomarker-negative status. There were no substantial differences in functional measures between individuals with biomarker-negative status and those with biomarker-positive status; there were only small differences in neuropsychological measures, which were not clearly clinically meaningful. Individuals with clinical dementia but absent plasma biomarkers for AD likely did not meet the A/T/N classification; this group may consist of those with non-AD dementias, early AD with low cognitive reserve, or mixed dementia. Supporting the presence of non-AD dementias is the observation that *APOE-ε4* genotype was associated with biomarker-positive AD. Thus, for clinical AD, the use of plasma biomarkers adds specificity to the diagnosis.

Individuals without dementia included a small proportion who had biomarker-positive status. It is likely that this group has incipient AD and may develop clinical symptoms over time. Many studies have shown that a proportion of elderly individuals may have positive AD biomarkers by PET or CSF, and these individuals are at increased risk of developing AD.^22^ It is likely that plasma biomarkers, like PET or CSF, might identify premorbid AD among otherwise healthy individuals without dementia. In that sense, the use of plasma biomarkers added sensitivity to the neuropsychological and functional assessment in this study.

The finding of increased levels of plasma GFAP among individuals with and without dementia who had plasma P-tau181 or P-tau181/Aβ42 biomarker-positive status is consistent with previous studies.^23,24,25^ The GFAP intermediate filament-III protein is found in several cell types, including astrocytes in the central nervous system, and correlates with Aβ plaque density. The plasma GFAP biomarker is also thought to be associated with brain amyloid-β pathology but not tau aggregation. In plasma, GFAP is associated with AD-related pathologies such as cerebral microbleeds and white matter hyperintensities. Increased GFAP was associated with elevated P-tau181 and P-tau181/Aβ42, regardless of the clinical diagnosis, indicating its potential role in identifying preclinical AD. We agree with Pereira et al,^26^ who proposed that plasma GFAP “should be incorporated in models of Alzheimer’s disease pathogenesis,” in that it may detect early astrocytosis secondary to amyloid-β pathology, but we would add consideration of elevated plasma P-tau181 and other biomarkers.

In this study, plasma NfL was also substantially higher among individuals with and without dementia who had P-tau181 levels greater than the diagnostic cut points. The NfL is a nonspecific biomarker of neuronal injury. Elevated NfL levels have been shown to be associated with cerebrovascular disease and independently with AD. Longitudinal studies have also shown that NfL and P-tau181 change over time with disease progression.^27,28^ However, as a stand-alone biomarker, NfL lacks specificity for AD-related diagnoses, although NfL levels may identify individuals who will develop cognitive decline.^29^

### Limitations

In considering the relative utility of plasma and CSF measurements, there are several limitations to consider. First, CSF is in contact with the brain and thus is more physically proximal to parenchymal pathological changes than plasma. Second, the biomarkers studied are at much lower concentrations in plasma than in CSF and thus are not as easily measured. Finally, plasma biomarkers may be more susceptible to systemic blood changes, including kidney function.^17^ To address biomarker concentrations, new techniques (including the Simoa assays used here) have allowed plasma measurements with reasonably good sensitivity and reproducibility. The proximity of CSF to brain tissue does not necessarily guarantee better biomarker performance because of different dynamics of protein entrance and clearance from fluids. Thus, it is not self-evident that CSF concentrations provide a better measurement of brain biomarkers than plasma, particularly earlier in the disease process.

This study has some limitations. Owing to its observational nature, it lacked neuroimaging by MRI, computed tomography, or PET, which are helpful for clinical diagnoses. The availability of traditional diagnostic tools is limited in the Dominican Republic. The ethnic distribution of the study group precludes generalization to other ethnic groups. However, the use of plasma-based biomarkers in this low-resource population from the Dominican Republic suggests a means to add both sensitivity and specificity to the clinical diagnosis of AD.

## Conclusions

There are several important conclusions to be drawn from this decision analytical modeling study. We observed an association between CSF and plasma biomarkers. In an observational research group such as this one, the opportunities for autopsy, amyloid PET, and CSF sampling may be limited, whereas plasma is readily available. While just over half of those with clinical AD had biological AD by plasma biomarker, the remaining individuals with clinical dementia need to be further investigated to determine the cause of their cognitive impairment. These findings alone support the use of plasma-based biomarkers as an adjunct to improve specificity of the diagnosis for observational and related clinical studies. Equally important was the observation that nearly one-fifth of individuals considered to be healthy and without dementia had biological evidence of AD. This finding suggests that plasma biomarkers such as PET and CSF may provide sensitive indicators of preclinical AD.

This study adds considerably to the previously published literature and supports the use of plasma-based biomarkers in observational and clinical studies as a cost-effective and practical method to add specificity to the clinical diagnosis. This approach is especially important when the acquisition of functional brain imaging and CSF sampling is limited. In addition, plasma biomarkers appear to provide a means to identify individuals in the premorbid states of AD.

## Notes

Supplement.
Data Sharing Statement
