# Supplementary material for: Evaluation of Plasma Biomarkers for A/T/N Classification of Alzheimer Disease Among Adults of Caribbean Hispanic Ethnicity
Source: JAMA Netw Open. 2023 Apr 20;6(4):e238214. doi: 10.1001/jamanetworkopen.2023.8214 (PMC10119732; doi:10.1001/jamanetworkopen.2023.8214)
Supplement: Supplement. — Data Sharing Statement [file jamanetwopen-e238214-s001.pdf]

Data Sharing Statement

Honig. Evaluation of Plasma Biomarkers for A/T/N Classification of Alzheimer Disease Among Adults of Caribbean Hispanic Ethnicity. *JAMA Netw Open*. Published April 20, 2023. doi:10.1001/jamanetworkopen.2023.8214

Data
Data available: Yes
Data types: Deidentified participant data, Data dictionary
How to access data: Data from this study are shared though the Columbia University Alzheimer Disease Center website: https://www.neurology.columbia.edu/research/research-centers-and-programs/alzheimers-disease-research-center-adrc/investigators/investigator-resources.
When available: beginning date: 04-01-2024

Supporting Documents
Document types: None

Additional Information
Who can access the data: researchers whose proposed use of the data has been approved
Types of analyses: purposes whose proposed use of the data has been approved
Mechanisms of data availability: no support provided; data available after approval of proposal; signed data access agreement required
